# The Impact of the COVID-19 Pandemic on the Psychological Well-being of Healthcare Workers in Obstetrics and Gynaecology: An Observational Study at an Apex Institute

**DOI:** 10.7759/cureus.24040

**Published:** 2022-04-11

**Authors:** Jyoti Meena, Soniya Dhiman, Renu Sharma, Kamlesh Kumari, Seema Singhal, Vidushi Kulshrestha, Richa Vatsa, Vanamail Perumal

**Affiliations:** 1 Department of Obstetrics and Gynaecology, All India Institute of Medical Sciences, New Delhi, New Delhi, IND; 2 Department of Psychiatry, All India Institute of Medical Sciences, New Delhi, New Delhi, IND; 3 Statistics and Demography, Department of Obstetrics and Gynaecology, All India Institute of Medical Sciences, New Delhi, New Delhi, IND

**Keywords:** covid-19, post-traumatic stress disorder, depression, mental health, health care workers

## Abstract

Background and objective

The coronavirus disease 2019 (COVID-19) pandemic has affected the health, social, and economic sectors all over the world. With a view to assessing the impact of COVID-19 on the mental health of healthcare workers (HCWs), we conducted a study to find out the incidence and severity of depression, anxiety, and post-traumatic stress disorder (PTSD) among HCWs.

Material and methods

This was an observational cross-sectional study conducted in the Department of Obstetrics and Gynaecology in collaboration with the Department of Psychiatry at AIIMS, New Delhi from March 2021 to June 2021. One hundred HCWs working in the Department of Obstetrics and Gynaecology were surveyed using a set of semi-structured interview schedules and structured questionnaires distributed via email or manually. The structured questionnaire included the demographic profile; other baseline information; the 42-item Depression, Anxiety, and Stress Scale (DASS-42); and the Impact of Event Scale-Revised (IES-R) instrument. Data analysis was carried out using the statistical package STATA version 14.0 (StataCorp LLC, College Station, TX).

Results

A total of 100 HCWs participated in the study, out of which 39 (39%), 45 (45%), and 16 (16%) were doctors, nursing staff, and supporting staff, respectively. Overall, 92 (92%) of the participants were women, and the mean age of the participants was 29.87 ±4.85 years. Out of the 100 participants, 17 (17%), 25 (25%), 13 (13%), and two (2%) participants had depression, anxiety, stress, and PTSD, respectively. Occupation-wise, among the nursing staff, doctors, and supporting staff, the incidence of depression was 24.4%, 15.4%, 0.0%, respectively; the anxiety rate was 33.3%, 25.6%, and 0.0%, respectively; and the rate of stress was 17.8%, 12.8%, and 0.0%, respectively. The IES-R score was significantly higher among unmarried as compared to married participants (2.70 ±7.935 vs. 1.60 ±3.583, p=0.000). Participants living in joint families had a higher DASS-42 score (DASS-D: 4.00 ±5.299 vs. 3.77 ±7.727, p=0.889; DASS-A: 4.31 ±4.398 vs. 4.12 ±7.496, p=0.905; DASS-S: 4.08 ±4.816 vs. 3.88 ±7.567, p=0.016) and lower IES-R score (1.31 ±4.922 vs. 2.66 ±9.947, p=0.752) as compared to those living in nuclear families. Depression (4.86 ±8.165 vs. 2.00 ±4.388, p=0.054), anxiety (5.31 ±7.538 vs. 2.14 ±4.704, p=0.024), stress (5.20 ±7.651 vs. 1.67 ±4.733, p=0.014) and PTSD (3.61 ±10.900 vs. 1.44 ±2.634, p=0.245) were all higher among HCWs having exposure to COVID-19 more than 10 hours per week compared to participants with an exposure of less than 10 hours per week. The participants having psychiatric illness in the family showed significantly higher mean values for DASS-42 (DASS-D: 20.00 ±26.870 vs. 3.50 ±6.264, p=0.001; DASS-A: 18.50 ±20.506 vs. 3.88 ±6.215, p=0.002; DASS-S: 18.00 ±21.213 vs. 3.64 ±6.346, p=0.003) as compared to those without any psychiatric illness in the family.

Conclusion

Based on our findings, occupational and environmental factors at the workplace play a key role in mental health outcomes, and COVID-19 has had a significant impact on the mental health of HCWs. Furthermore, we have also observed that effective planning can significantly reduce mental stress.

## Introduction

In December 2019, the world witnessed the emergence of several cases of pneumonia of unknown cause in China, which eventually led to a pandemic of global proportions called coronavirus disease 2019 (COVID-19), caused by severe acute respiratory syndrome coronavirus 2 (SARS-CoV-2). The WHO declared COVID-19 a global pandemic in March 2020 [1,2]. The first case in India was reported in January 2020 from Kerala [3]. Since then, several cases have been reported, and soon the virus spread throughout the country, with disastrous consequences for the health, social, and economic sectors in India. As suggested by previous studies [4,5], healthcare workers (HCWs), who are at the forefront of the fight against COVID-19, are always at a risk of mental health crises during an infectious disease outbreak. However, the COVID-19 pandemic is associated with some specific characteristics that increased its capacity to impact the mental health of HCWs. Firstly, the highly infectious nature of the virus; the fact that the pandemic has affected millions of people in several countries has led to colossal anxiety among HCWs with regard to concerns related to the safety of their own and that of their loved ones. Second, the changes in working dynamics; the highly infectious nature of the disease, the way it has spread, the focus on the use of personal protective equipment (PPE), and the frequently changing guidelines have all imposed a change in the working dynamics of the hospitals as well as the concerned departments.

At our institute also, following a concerted effort by the department in collaboration with hospital administration, we prepared ourselves to face the challenges posed by COVID-19. A road map for the new normal was developed and implemented to smoothen the obstetric care to be provided during COVID-19 as early as possible [6]. The working policies have been constantly updated, implemented, and tested, and then actions have been taken according to evolving situations. These repeated alterations, in an already hectic department, created a higher level of mental and physical stress among the residents, faculty, and other healthcare staff. In addition to this, repeatedly changing duty rosters, working in PPE, social distancing, not being able to meet the family members, concerns for families' safety, and the extended periods of isolation and quarantine have all impacted the mental health of HCWs.

Various studies have shown that this pandemic has profoundly affected the mental health of HCWs, and many doctors and nurses have been suffering from depression, anxiety, and increased levels of stress [7-11]. High levels of stress and anxiety have been shown to reduce staff morale, increase absenteeism from the workplace, and lower work satisfaction and quality of care [5,12]. Hence, it is critical to understand the psychological needs of HCWs in order to provide them with the optimal support to overcome the effect of the COVID-19 pandemic. Studies have clearly shown that while HCWs are a group highly vulnerable to negative psychological distress, the impact is not the same for all segments of HCWs [8]. Therefore, there is a need to identify the subgroups within this population to target their specific psychological needs and provide support accordingly.

This study aimed to assess the incidence and severity of depression, anxiety, and post-traumatic stress disorder (PTSD) among HCWs and also to explore the association of these with background variables including gender, marital status, occupation, medical history, and level of involvement with COVID-19 patients. Additionally, we aimed to identify the more vulnerable subgroups and the potential risk factors so that early intervention can be planned and continuity of care maintained.

## Materials and methods

This was an observational cross-sectional study conducted in the Department of Obstetrics and Gynaecology in collaboration with the Department of Psychiatry at AIIMS, New Delhi from March 2021 to June 2021 after obtaining ethical approval from the ethics committee of the institute. We enrolled 100 HCWs working in the Department of Obstetrics and Gynaecology, including doctors, nursing staff, and supporting staff members. HCWs of both genders who were willing to participate were included in the study. Those having any prior history of depression, anxiety, psychosis, or other psychological problems were excluded from the study.

Data collection

The survey was conducted using a set of semi-structured interview schedules, and structured questionnaires were distributed via email or manually as per the convenience of the participants. The semi-structured questionnaire schedule sought information regarding the demographic profile of the participants, including age, gender, religion, educational level, relationship status, occupational history, medical history, level of direct involvement with COVID-19 patients, the source of information regarding COVID-19, and whether the training provided by the institute had helped them or not. The structured questionnaire included the Depression, Anxiety, and Stress Scale (DASS-42) [13], which consisted of 42 items, with each of the three DASS-42 sub-scales containing 14 items, and the Impact of Event Scale-Revised (IES-R) instrument [14], which is a self-reported questionnaire comprising 22 questions rated on a 4-point scale on the number of symptoms one had experienced in the preceding week. Both the questionnaires are freely available in the public domain. The three scales include intrusion, avoidance, and hyperarousal. The first two domains contain eight items, and the third one has six items. The IES-R is recognized as one of the first self-report tools developed to assess post-traumatic stress [14]. For outcome purposes, the score interpretation for both the instruments was done according to their standards. The scores of >10, >7, and >14 were considered for depression, anxiety, and stress, which were further classified into mild, moderate, severe, and extremely severe. For IES-R, a score >24 was considered to be of clinical relevance for PTSD.

Statistical analysis

Data analysis was carried out using the statistical package STATA version 14.0 (StataCorp LLC, College Station, TX). All questions were given numerical codes and the answers were categorized into mild, moderate, and severe according to the cut-off value for each domain. Descriptive measures such as mean, standard deviation (SD), and range values were calculated for each domain and compared across the categories using the analysis of variance (ANOVA) test. The association of the severity of the anxiety, depression, and stress level with background variables was compared using Pearson's chi-squared test. A two-sided probability of p<0.005 was considered statistically significant for all statistical tests.

## Results

A total of 100 HCWs participated in the study, out of which 39 (39%) were doctors, 45 (45%) were nursing staff, and 16 (16%) were supporting staff, including health assistants, technicians, guards, and sanitary assistants. Overall, 92 (92%) of the participants were women, and the mean age of the participants was 29.87 ±4.85 years. Among the cohort, 70 (70%) were graduates, 46 (46%) were married and living with their families, and 74 (74%) had nuclear families (Table 1).

We also studied other parameters to assess the risk factors and protective factors. Almost all of the participants agreed that they received most of the information regarding COVID-19 from the institute only. The training and facilities provided by the institute were very satisfactory; specifically, the training regarding the proper use of the PPE kit and the segregation and infection control measures were very valuable and helped them a lot to be confident and safe while performing their duties as a frontline worker. All the participants had full family support, and fear of the family's well-being bothered all of them. Our participants were very motivated and trained and were not afraid of getting an infection or bothered by the social stigma and fear of any isolation or quarantine (Table 1).

All HCWs agreed that our system's policy of shorter working hours in the COVID-19 area, segregated teams for different areas, rotating shifts, and regular breaks helped boost their morale and enhanced their working efficiency. Hence, in the limited period of this study, some had more exposure to COVID-19 patients than others. In our study, 36 (36%) participants had less than 10 hours/week of exposure, and 64 (64%) participants had more than 10 hours/week of exposure to COVID-19-infected patients (Table 1).

**Table 1:** Demographic profile of the participants COVID-19: coronavirus disease 2019; SD: standard deviation

Characteristics	Overall (n=100)	Doctors (n=39)	Nursing staff (n=45)	Supporting staff (n=16)
Age (years), mean ±SD	29.87 ±4.855	27.59 ±2.500	30.89 ±5.055	32.56 ±6.356
Gender, n (%)				
Female	92 (92%)	37 (94.8%)	45 (100%)	10 (62.5%)
Male	8 (8%)	2 (5.2%)	0	6 (37.5%)
Educational level, n (%)				
Graduate	70 (70%)	18 (46.2%)	42 (93.3%)	10 (62.5%)
Postgraduate	24 (24%)	21 (53.8%)	3 (6.7%)	0
Others	6 (6%)	0	0	6 (37.5%)
Marital status, n (%)				
Married	46 (46%)	13 (33.3%)	24 (53.3%)	10 (62.5%)
Unmarried	54 (54%)	26 (66.7%)	21 (46.7%)	6 (37.5%)
Type of family, n (%)				
Joint	26 (26%)	9 (23.1%)	14 (31.1%)	3 (18.8%)
Nuclear	74 (74%)	30 (76.9%)	31 (68.9%)	13 (81.2%)
Family support, n (%)				
Yes	100 (100%	39 (100%)	45 (100%)	16 (100%)
Fear of family well-being, n (%)				
Yes	Present in all (100%)	Present in all (100%)	Present in all (100%)	Present in all (100%)
Received COVID-19 prevention training, n (%)				
Yes	100 (100%)	39 (100%)	45 (100%)	16 (100%)
Training helped, n (%)				
Yes	100 (100%)	39 (100%)	45 (100%)	16 (100%)
Interaction with COVID-19 patients, n (%)				
<10 hours/week	36 (36%)	1 (2.6%)	24 (53.3%)	11 (68.8%)
>10 hours/week	64 (64%)	38 (97.4%))	21 (46.7%)	5 (31.2%)
Fear of social stigma, quarantine, or COVID-19 area duty, n (%)	0 (0%)	0 (0%)	0 (0%)	0 (0%)

On evaluating the psychosocial aspect of the participants, we found that out of 100, 17 (17%), 25 (25%), 13 (13%), and two (2%) participants had depression, anxiety, stress, and PTSD respectively. Occupation-wise, among the nursing staff, doctors, and supporting staff respectively, the incidence of depression (24.4% vs. 15.4% vs. 0.0%, p=0.154), anxiety (33.3% vs. 25.6% vs. 0.0%, p=0.030), and stress (17.8% vs. 12.8% vs. 0.0%, p=0.192) were higher among the nursing staff compared to doctors and supporting staff, but this difference was not statistically significant (Table 2).

**Table 2:** Psychological well-being of the participants in terms of depression, anxiety, stress, and PTSD *Pearson's chi-squared test was applied PTSD: post-traumatic stress disorder

Variables	Doctors, n (%)	Nursing staff, n (%)	Supporting staff, n (%)	P-value*
Depression	6 (15.4%)	11 (24.4%)	0 (0.0%)	0.154
Anxiety	10 (25.6%)	15 (33.3%)	0 (0.0%)	0.030
Stress	5 (12.8%)	8 (17.8%)	0 (0.0%)	0.192
PTSD	1 (2.6%)	1 (2.2%)	0 (0.0%)	0.818

None of the male participants showed any feature of depression, anxiety, stress, or PTSD; however, due to the small number of male participants, the difference was not found to be statistically significant (Table 3).

**Table 3:** Psychological well-being on the basis of gender *Pearson's chi-squared test was applied PTSD: post-traumatic stress disorder

Variables	Female (n=92)	Male (n=8)	P-value*
Depression	17	0	0.496
Anxiety	25	0	0.145
Stress	13	0	0.329
PTSD	2	0	0.718

All the scoring tools mentioned above were compared with the demographic characteristics, including gender, marital status, family type, occupation, exposure to COVID-19 patients, and any chronic medical and psychological illness in the family. The female participants had a higher mean value for the DASS-42 score (DASS-D: 4.03 ±7.373 vs. 1.50 ±3.117, p=0.339; DASS-A: 4.43 ±7.014 vs. 1.13 ±2.031, p=0.1888; DASS-S: 4.17 ±7.139 vs. 1.13 ±2.800, p=0.235) and IES-R score (3.04 ±9.246 vs. 0.38 ±0.744, p=0.419) than males; but due to a minimal proportion of males in the study, the difference was not statistically significant. The IES-R score was significantly higher in unmarried as compared to married participants (2.70 ±7.935 vs. 1.60 ±3.583, p=0.000). Participants living in joint families had a higher DASS-42 score (DASS-D: 4.00 ±5.299 vs. 3.77 ±7.727, p=0.889; DASS-A: 4.31 ±4.398 vs. 4.12 ±7.496, p=0.905; DASS-S: 4.08 ±4.816 vs. 3.88 ±7.567, p=0.016) and lower IES-R score (1.31 ±4.922 vs. 2.66 ±9.947, p=0.752) as compared to those with nuclear families. The mean value of the DASS-42 score (DASS-D: 4.79 ±9.350 vs. 4.04 ±5.807 vs. 0.88 ±2.277, p=0.176; DASS-A: 5.08 ±8.459 vs. 4.56 ±6.066 vs. 0.88 ±1.544, p=0.100; DASS-S: 5.33 ±8.093 vs. 3.82 ±6.709 vs. 0.81 ±1.974, p=0.088) and IES-R score (3.95 ±10.763 vs. 2.78 ±8.628 vs. 0.25 ±0.447, p=0.378) was higher among doctors than nursing staff and supporting staff respectively, but the difference was not statistically significant. Depression (4.86 ±8.165 vs. 2.00 ±4.388, p=0.054), anxiety (5.31 ±7.538 vs. 2.14 ±4.704, p=0.024), stress (5.20 ±7.651 vs. 1.67 ±4.733, p=0.014), and PTSD (3.61 ±10.900 vs. 1.44 ±2.634, p=0.245) were all higher among HCWs having exposure to COVID-19 for more than 10 hours per week compared to participants having exposure for less than 10 hours per week. The participants having psychiatric illness in the family showed significantly higher mean values for DASS-42 (DASS-D: 20.00 ±26.870 vs. 3.50 ±6.264, p=0.001; DASS-A: 18.50 ±20.506 vs. 3.88 ±6.215, p=0.002; DASS-S: 18.00 ±21.213 vs. 3.64 ±6.346, p=0.003) as compared to those without any psychiatric illness in the family (Table 4).

**Table 4:** Correlation of DASS-42 and IES-R score with baseline characteristics *ANOVA test was applied ANOVA: analysis of variance; DASS: Depression, Anxiety, and Stress Scale; IES-R: Impact of Event Scale-Revised; SD: standard deviation

Characteristics	DASS-D (mean ±SD)	P-value*	DASS-A (mean ±SD)	P-value*	DASS-S (mean ±SD)	P-value*	IES-R (mean ±SD)	P-value*
Gender								
Male (n=8)	1.50 ±3.117	0.339	1.13 ±2.031	0.188	1.13 ±2.800	0.235	0.38 ±744	0.419
Female (n=92)	4.03 ±7.373		4.43 ±7.014		4.17 ±7.139		3.04 ±9.246	
Marital status								
Married (n=46)	3.78 ±5.456	0.180	4.16 ±4.734	0.122	3.67 ±5.617	0.045	1.60 ±3.583	0.000
Unmarried (n=54)	3.63 ±8.215		3.93 ±8.016		3.83 ±7.620		2.70 ±7.935	
Type of family								
Joint (n=26)	4.00 ±5.299	0.889	4.31 ±4.398	0.905	4.08 ±4.816	0.016	1.31 ±4.922	0.752
Nuclear (n=74)	3.77 ±7.727		4.12 ±7.496		3.88 ±7.567		2.66 ±9.947	
Occupation								
Doctor (n=39)	4.79 ±9.350	0.176	5.08 ±8.459	0.100	5.33 ±8.093	0.088	3.95 ±10.763	0.378
Nursing staff (n=45)	4.04 ±5.807		4.56 ±6.066		3.82 ±6.709		2.78 ±8.628	
Supporting staff (n=16)	0.88 ±2.277		0.88 ±1.544		0.81 ±1.974		0.25 ±0.447	
Exposure to COVID-19 patients/week								
>10 hours (n=64)	4.86 ±8.165	0.054	5.31 ±7.538	0.024	5.20 ±7.651	0.014	3.61 ±10.900	0.245
<10 hours (n=36)	2.00 ±4.388		2.14 ±4.704		1.67 ±4.733		1.44 ±2.634	
Presence of psychiatric illness in the family								
Absent (n=98)	3.50 ±6.264	0.001	3.88 ±6.215	0.002	3.64 ±6.346	0.003	2.81 ±8.986	0.852
Present (n=2)	20.00 ±26.870		18.50 ±20.506		18.00 ±21.213		4.00 ±000	
Presence of any medical illness in the family								
Absent (n=70)	4.11 ±6.875	0.546	4.66 ±6.737	0.276	4.50 ±6.848	0.211	2.64 ±7.398	0.102
Present (n=30)	3.17 ±7.839		3.03 ±6.946		2.60 ±7.069		3.27 ±11.820	

In terms of the severity of depression, out of 17, mild, moderate, severe, and extremely severe depression was present in eight (47.1%), four (23.5%), three (17.6%), and two (11.8%) participants respectively. When the severity of anxiety was observed, mild, moderate, severe, and extremely severe anxiety was found in 12 (48%), six (24%), three (12%), and four (16%) participants out of 25. With regard to stress, out of 13, six (46.2%), four (30.8%), and three (23.1%) were having mild, moderate, and severe stress. None of them had extremely severe stress. Two participants were having extremely severe PTSD.

## Discussion

The COVID-19 pandemic is an ongoing global challenge, and as the disease spreads rapidly, the most affected countries are facing huge challenges to fulfill the demands for PPE kits and infrastructure. However, India witnessed the emergence of the COVID-19 pandemic slightly later than the rest of the world, and the strict initial strategies implemented by the Government of India, like complete lockdown, had delayed the spread of the disease and provided sufficient time to our healthcare system to be prepared for the pandemic.

In our department, our proactive team took timely decisions and helped us prepare ourselves for handling this pandemic with the administration's help. Measures such as the sufficient supply of PPE kits, appropriate segregation of patients and treating HCWs in COVID-19-positive, suspect, and regular areas, continued provision of information regarding COVID-19, the training regarding the proper use of PPE kits, and other safety measures enabled our team to be prepared effectively and efficiently during COVID-19. This adequate level of preparation and the lower number of participants must be the reason that our results are not in concordance with the results of most of the studies conducted outside or inside India. In our study, the incidence of depression, anxiety, stress, and PTSD was 17%, 25%, 13%, and 2%, respectively, which was much lower than many international and national studies, as shown in one latest study where out of 1,563 HCWs, 50.7%, 44.7%, and 36.1% showed symptoms of depression, anxiety, and sleep deprivation, respectively [15]. A study done by Siddiqui et al. [16] demonstrated a significantly increased prevalence of anxiety among HCWs during the COVID-19 pandemic, and the primary reason for their anxiety was a lack of PPE and inadequate support. However, in our institute, proper training and facilities provided to the HCWs and the adequate availability of PPE kits explain the lower incidence of depression, anxiety, and stress compared to other studies.

Zhang et al. [9] and Lu et al. [10] have reported that medical HCWs have significantly higher levels of anxiety, depression, somatization, and OCD symptoms compared to non-medical HCWs. Our study also showed that supporting staff has not been as entirely affected by depression, anxiety, stress, and PTSD as compared to doctors and nursing staff. The reason could be that the supporting staff of our department was not posted in the COVID-19 pool; however, they do come across COVID-19-positive patients who were attended in the department and later shifted to COVID-19-designated center when they turned out to be COVID-19-positive. However, the exposure time was less compared to doctors and nurses. Besides this confidence related to safety, early training and social support from family members, team members, and the institute may also be factors responsible for this observation as indicated by previous studies [17-19]. However, the study by Tan et al. [11] has shown some contradictory results, in that non-medical HCWs have a higher prevalence of anxiety than their medical counterparts. Between the different medical HCW groups, various studies have shown that nurses were at risk of worse mental health outcomes than doctors [20-22]. Similarly, our study also found that nursing staff had higher levels of depression, anxiety, and stress than doctors. However, comparing the mean values, all the scores were higher among doctors than nurses, but the difference was not statistically significant. This could be attributed to the small sample size.

Being a frontline worker and the duration of exposure also affected the outcomes. At our institute, we had posted the staff on a rotation basis, and every participant was directly exposed to the patients; however, there was a difference in exposure time depending upon their ward postings. The participants with more than 10 hours per week of exposure to COVID-19 patients were affected more than the participants with less than 10 hours per week of exposure, and this difference was statistically significant. This finding is in line with the results shown in many other studies [21,23,24]. All the participants were concerned about their family's safety, and assurance regarding this was a tremendous stress-relieving factor. Cai et al. [20] have brought this up as the main stress factor, and it has been described in other previous studies as well [21,24,25].

The female gender has been considered a risk factor for developing depression, anxiety, and insomnia by Lai et al. [22]. Another large nationwide Chinese study has also shown similar results [9]. Similarly, in our study, the mean value of DASS-D, DASS-A, DASS-S, and IES-R scores was higher among females than males, although there was no statistically significant difference due to the smaller sample size and the big difference in the male-female ratio (8:92). Having an underlying psychiatric illness in the family significantly affected participants' mental health, and in our study, we have observed a statistically significant difference. Many studies have identified that having an organic illness in the family or the individual increases psychological distress and works as an independent risk factor [9].

When we considered the protective factors, almost all HCWs agreed that the provision of adequate PPE kits, good hospital guidance, and the facilities provided to them were very satisfactory and helped them maintain their mental stability during this pandemic. In addition, measures such as continuous training in the form of mock drills, segregation of all the wards and the pathways heading to the COVID-19 areas, the sterilization system, contact-tracing of affected persons, and provision of appropriate isolation and quarantine facilities for HCWs and their families ensured the HCWs that the institute is keenly concerned regarding their safety. Various studies have also shown that good hospital guidance, increased knowledge about the disease, and proper training reduce the stress experienced by the HCWs [20,26,27]. Similar to other studies, our study has also shown that other factors such as being married, having family support, and good team coordination were very effective in reducing stress [28,29]. Studies have also suggested that shorter working hours, regular breaks, and rotating shifts are reliable methods to boost the morale of HCWs, and our institute followed the same.

The major strength of our study is that we had included participants from all segments of HCWs, including doctors, nursing staff, health attendants, sanitary attendants, and technicians, and conducted a subgroup analysis to find out the most vulnerable group in terms of psychological impact so that appropriate counseling and management can be provided. In addition, many previous studies did not address the medical history of psychiatric disorders in the family or the participant itself; we have included both and excluded participants who had any form of psychiatric illness.

This study has some limitations. Firstly, it was a self-reported survey, and this may have led to recall bias. Our sample size was very small and represented only a small group of our team, especially the supporting staff, which may have affected the results. The study was conducted at an apex institute, and due to the lack of generalized SOPs, the management system can be different at different institutes; hence the results do not apply to the whole population of HCWs working in the obstetrics and gynaecology departments in the country. The study was conducted during the end of the first wave of COVID-19 when the HCWs had become more prepared mentally and socially. The first wave did not have so much impact and this factor can impair the generalizability of results. Finally, we did not have any other data to compare our results against, such as those collected during pre-COVID-19 or post-COVID-19 periods; hence, it is not easy to ascertain if the psychological impact is associated with COVID-19 alone or if any other factors also played a role.

Our study clearly showed that even after providing adequate facilities to the HCWs, their mental stress levels were very high, and we need to take more action to make the environment more comfortable for our HCWs. We can be exposed to this type of situation again; hence, we should be prepared to overcome the stress related to such circumstances in the near future and should put in place proper facilities and resources for our HCWs so that they can deal with the challenges effectively.

## Conclusions

Our study showed that occupational and environmental factors at the workplace play a key role in mental health outcomes, and COVID-19 has significantly impacted the mental health of HCWs. Adequate planning, complete and timely provision of information, proper provision of PPE kits, along with regular training of the staff significantly contributed to reducing their mental stress at our institute. We may encounter similar outbreaks and pandemics in the future as well, and hence to protect and improve the psychological well-being of the HCWs who are often at the forefront of fighting these events, we can initiate screening programs and can explore various psychotherapeutic interventions and support mechanisms for HCWs.
